# Distribution of CCR5-Delta32, CCR2-64I, and SDF1-3’A host genetic factors in HIV-infected and uninfected individuals in Luanda, Angola

**DOI:** 10.1186/s12981-025-00751-7

**Published:** 2025-05-24

**Authors:** Cruz S. Sebastião, Victor Pimentel, Domingos Jandondo, Joana M.K. Sebastião, Euclides Sacomboio, Marta Pingarilho, Miguel Brito, Edson Kuatelela Cassinela, Jocelyne Neto de Vasconcelos, Ana B. Abecasis, Joana Morais

**Affiliations:** 1 Centro Nacional de Investigação Científica (CNIC), Luanda, Angola; 2 Centro de Investigação em Saúde de Angola (CISA)|Instituto Nacional de Investigação em Saúde (INIS), Luanda, Angola; 3 Global Health and Tropical Medicine, Associate Laboratory in Translation and Innovation Towards Global Health, GHTM, LA-REAL, Instituto de Higiene e Medicina Tropical, IHMT, Universidade NOVA de Lisboa, UNL, Rua da Junqueira 100, Lisboa, 1349-008 Portugal; 4 Instituto de Ciências da Saúde (ICISA), Universidade Agostinho Neto (UAN), Luanda, Angola; 5 H&TRC - Health & Technology Research Center, ESTeSL - Escola Superior de Tecnologia da Saúde, Instituto Politécnico de Lisboa, Lisbon, Portugal

**Keywords:** HIV, AIDS-related gene variants, Allelic frequency, Angola

## Abstract

**Background:**

The HIV/AIDS pandemic remains a public health concern. Studies on host genetic polymorphisms that confer resistance to HIV-1 infection or delay HIV disease progression are scarce in African countries. Herein, we investigate the proportion of the mutated phenotype of the AIDS-related polymorphisms CCR5-Delta32, CCR2-64I, and SDF1-3’A in HIV-infected and uninfected individuals in Luanda, the capital of Angola, a sub-Saharan African country.

**Methods:**

This was a cross-sectional study conducted with 284 individuals, of whom 159 were HIV-negative and 125 were HIV-positive. The CCR5-Delta32, CCR2-64I, and SDF1-3′A genotypes were detected by conventional PCR and visualised on 2% agarose gel. A Chi-square test determined the frequency of each genetic variant and was deemed significant when *p* < 0.05.

**Results:**

The frequency of CCR5-Delta32, CCR2-64I, and SDF1-3 A was 0% (0/272), 60.2% (154/256), and 42.5% (114/268), respectively. CCR2-64I and SDF1-3 A polymorphisms were statistically related to HIV infection (*p* < 0.001). Statistically significant was observed between ABO blood groups (*p* = 0.006) and HIV-1 subtype (*p* = 0.015) with CCR2-64I. Also, the age group (*p* = 0.024) and RH blood group (*p* = 0.018) were statistically related to the distribution of SDF1-3 A polymorphism.

**Conclusions:**

We found no CCR5-Delta32 allele, while CCR2-64I and SDF1-3’A were found and presented a relationship with HIV infection, age, ABO/RH blood group, and HIV-1 subtypes. The observed associations of CCR2-64I and SDF1-3′A with HIV underscore the urgent need for further multidisciplinary research, with potential implications for targeted prevention and public health strategies. Therefore, studies investigating biological and non-biological factors related to susceptibility to HIV infection and AIDS progression or death should be conducted in Angola.

## Background

The HIV/AIDS pandemic remains a significant and ongoing public health challenge with considerable global implications [1]. To identify critical host factors that facilitate HIV replication, there has been increasing research into candidate genes associated with HIV infection and AIDS progression or death [2]. Despite this progress, there is still a lack of extensive data on the distribution and prevalence of host genetic polymorphisms that may confer resistance to HIV-1 infection or delay the progression of HIV-related diseases in African populations [3]. This knowledge gap is particularly significant in sub-Saharan Africa, where the burden of HIV/AIDS remains high. Therefore, investigating the allelic variations of important genetic polymorphisms, such as CCR5-Delta32, CCR2-64I, and SDF1-3′A, as well as their distribution across populations, may provide crucial insights into the HIV-1 infection epidemiology, disease progression, and its clinical management [4].

The CCR5-Delta32 mutation has attracted attention because of its association with resistance to HIV infection in individuals who are homozygous for the mutation (mt/mt) [5–7], although other studies showed that some HIV individuals harbour the CCR5-Δ32/Δ32 genotype [8, 9]. CCR2-64I mutation, involving a valine-to-isoleucine substitution at position 64 of the CCR2 receptor, does not appear to affect initial susceptibility to HIV infection, individuals who are heterozygous (wt/mt) or homozygous (mt/mt) for this mutation tend to experience slower disease progression possibly due to delayed viral replication, with delayed onset of AIDS or prolonged survival [10]. Individuals homozygous (mt/mt) for the SDF1-3′A mutation, a polymorphism within the SDF1 gene, exhibit faster disease progression, suggesting a potential role for this genetic variant in accelerating HIV-1 pathogenesis [11–14].

These AIDS-related genetic polymorphisms (ARGs) have been shown to modulate susceptibility to HIV-1 infection and/or progression to AIDS [15]. However, the precise influence of these genetic variants on HIV susceptibility and disease progression remains controversial [3, 4]. Given the complexity of these host genetic factors and their potential clinical implications, a better understanding of their distribution in diverse populations is crucial. Currently, there is no study published assessing the host genetic polymorphisms that confer resistance to HIV-1 infection or delay HIV disease progression in Angola. This study aimed to investigate the proportion of the mutated phenotype of the main AIDS-related polymorphisms, CCR5-Delta32, CCR2-64I, and SDF1-3′A among HIV-infected and uninfected individuals in Luanda, the capital city of Angola, a country located in sub-Saharan Africa, in order to contribute to an understanding of the host genetic factors that influence HIV susceptibility and progression in this population and thus provide valuable information to adapt preventive and therapeutic strategies for the management of HIV/AIDS in Angola and similar regions in Africa.

## Methods

### Study design and setting

This was a part of a larger cross-sectional study, in which 284 individuals were randomly selected for inclusion. Of which, 159 were HIV-negative and 125 HIV-positive selected at the National Blood Transfusion Service in Angola (NBTS), a reference health unit, located in Luanda, the capital city of Angola, between March to May 2022. We included adults, male or female, and all those recently diagnosed with HIV had not been exposed to ART. The study was conducted following Helsinki’s Declaration. This study was approved by the National Ethics Committee of the Angolan Ministry of Health (nr. 39/2021, approved on 01 December 2021) and the direction board of the National Blood Transfusion Service (nr.128/GDG/INS/2022, approved on 24 February 2022). Participants were informed about the objectives of the study, and verbal informed consent was obtained from all enrolled participants even before they were considered part of the study.

### Data/sample collection and laboratory procedure

All the participants were screened for anti-HIV (Abbott, USA) with the ARCHITECT Plus i2000sr (Abbott, USA), following the manufacturer’s instructions. HIV-reactive and non-reactive participants were randomly selected and included in the test (HIV+) and control (HIV-) groups, respectively. HIV-1 viral load was determined using the Applied Biosystems m2000rt Real Time PCR Instrument, following the manufacturer’s protocols. We used a questionnaire to collect sociodemographic data, such as age and gender, from the participants. Also, 5 ml of an intravenous whole blood sample was collected into tubes containing ethylenediaminetetraacetic acid (EDTA). ABO/Rh blood group phenotypes determination was carried out using blood reagents and diagnostic kits (Lorne Laboratories Limited, UK), following the manufacturer’s instructions [16]. HIV-1 genotyping and bioinformatic analysis were performed as described by Sebastião et al., 2024 [17, 18]. REGA [19] and Comet [20] genotyping tools were used to examine the HIV-1 genetic diversity. Calibrated Population Resistance (CPR) tool (https://hivdb.stanford.edu/cpr/) was used to calculate the proportions of individuals with NRTI, NNRTI, PI, and INSTI-associated resistance, considering the list published by Tzou et al., 2020 [21].

Furthermore, blood samples were collected from all participants, spotted on DBS Whatman 903, and allowed to dry at room temperature.

### Genotyping using DBS samples

DNA was extracted from DBS using the QIAamp DNA Mini Kit (Qiagen) following the manufacturer’s instructions. The CCR5-Delta32, CCR2-64I and SDF1-3′A genotypes were detected by conventional PCR with specific primers covering polymorphic sites of CCR5-Delta32 (forward– 5′-CTTCATCATCCTCCTGACAATCG‐3′ and reverse– 5′‐GACCAGCCCCAAGTTGACTATC‐3′), SDF1-3 A (forward– 5′‐CAGTCAACCTGGGCAAAGCC‐3′ and reverse– 5′‐AGCTTTGGTCCTGAGAGTCC‐3′), and CCR2-64I (forward– 5′‐GGATTGAACAAGGACGCATTTCCCC‐3′ and reverse– 5′‐TTGCACATTGCATTCCCAAAGACCC‐3′) as described by Nkenfou et al., 2013 [22]. For the PCR reaction, 5µL (ranging 1.5–19ng) of extracted DNA was used in a mixture of a final volume of 20µL containing 12.5µL of GoTaq^®^ Green Master Mix (PROMEGA) or NZYTaq II 2x Green Master Mix (NZYTECH), 2.5µL of forward primer, 2.5µL of reverse primer, and 2.5µL of ultrapure water. The mixture was run in a GeneAmp PCR System 2700/9700 thermocycler (Applied Biosystems) at 95 °C for 5 min followed by 35 cycles of 95 °C for 45 s, 58/63°C (annealing temperature) for 45 s, and 72 °C for 45 s, and a final extension of 72 °C for 10 min. The annealing temperature of 58 °C was the same for CCR5-Delta32, SDF1-3 A, while for the CCR2-64I gene, it was 63 °C. PCR products were visualised on 2% agarose gel. Primers CCR5-Delta32 produce a 262 bp fragment if the person does not have the mutation on both alleles (wild type), while CCR2-64I produces a 380 bp fragment and SDF1-3 A produces a 302 bp fragment.

### Statistical analysis

The analysis was conducted in SPSS version 29 (IBM SPSS Statistics, USA). The descriptive analysis was presented as frequencies and percentages. The differences in the frequency of each genetic variant between HIV-1 seronegative and HIV-1 seropositive groups, which were our dependent variables, were determined by a Chi-square or Fisher’s exact test, when appropriate. A value of *P* < 0.05 was considered statistically significant.

## Results

### Distribution of CCR5-Delta32, CCR2-64I and SDF1-3’A host molecular factors

The demographic characterisation and distribution of host genetic factors CCR5-Delta32, CCR2-64I, and SDF1-3’A related to HIV infection and progression are presented in Table 1. Of the 284 enrolled participants, 10.9% (31/284) were female, while 89.1% (253/284) were male. Ages ranged from 19 to 60 years old, with a mean of 33±9.4 years. The age group of 20 to 40 years was predominant with 75% (213/284), followed by participants over 40 years (22.2%, 63/284). Regarding HIV infection status, the population consisted of 159 (56%) HIV-negative and 125 (44%) HIV-positive. The frequency of CCR5-Delta32, CCR2-64I, and SDF1-3 A polymorphisms was 0% (0/272), 60.2% (154/256), and 42.5% (114/268), respectively. CCR2-64I and SDF1-3 A polymorphisms were statistically related to HIV infection (*p* < 0.001). The presence of the CCR2-64I polymorphism in the heterozygous form (wt/mt) was 79.5% in HIV-uninfected individuals which corresponds to 3.9 times more than that observed in positive individuals, while in the homozygous form with the mutant allele (mt/mt), it was observed in 93.4% in HIV-uninfected individuals and only 6.3% in HIV-infected individuals, which corresponds to 14.9 times more than that observed in positive individuals (*p* < 0.001). On the other hand, the SDF1-3 A polymorphism, whether in the heterozygous (wt/mt) or homozygous (mt/mt) form with the mutant allele, was more frequent in HIV-positive individuals (*p* < 0.001). Among HIV-positive individuals, the proportion of SDF1-3′A was approximately 37.7 times higher in the heterozygous form and about 10 times higher in the homozygous form, compared to HIV-negative individuals.

**Table 1:** Distribution of CCR5-Delta32, CCR2-64I, and SDF1-3’A host molecular factors in HIV-infected and uninfected individuals in Luanda, Angola

Gene variants	*N* (%)	Gender			Age group				HIV status		
		Female (%)	Male (%)	*p*-value	< 20 yrs (%)	20–40 yrs (%)	> 40 yrs (%)	*p*-value	Negative (%; 95% CI)	Positive (%; 95% CI)	*p*-value
Overall	284 (100)	31 (10.9)	253 (89.1)		8 (2.8)	213 (75.0)	63 (22.2)		159 (56.0)	125 (44.0)	
CCR5-Delta32 (%)	272 (100)	29 (10.7)	243 (89.3)		8 (2.9)	205 (75.4)			147 (54.0; 45.9–62.1)	125 (46.0; 37.3–54.7)	
wt/wt	272 (100)	29 (10.7)	243 (89.3)	-	8 (2.9)	205 (75.4)	59 (21.7)	-	147 (54.0; 45.9–62.1)	125 (46.0)	-
wt/mt	0 (0.0)	0 (0.0)	0 (0.0)		0 (0.0)	0 (0.0)	0 (0.0)		0 (0.0)	0 (0.0)	
mt/mt	0 (0.0)	0 (0.0)	0 (0.0)		0 (0.0)	0 (0.0)	0 (0.0)		0 (0.0)	0 (0.0)	
CCR2-64I (%)	256 (100)	28 (10.9)	228 (89.1)		8 (3.1)	189 (73.8)	59 (23.0)		133 (52.0; 43.5–60.5)	123 (48.0; 39.2–56.8)	
wt/wt	102 (39.8)	12 (11.8)	90 (88.2)	0.922	1 (1.0)	75 (73.5)	26 (25.5)	0.192	6 (5.9)	96 (94.1)	**< 0.001**
wt/mt	122 (47.7)	13 (10.7)	109 (89.3)		4 (3.3)	91 (74.6)	27 (22.1)		97 (79.5)	25 (20.5)	
mt/mt	32 (12.5)	3 (9.4)	29 (90.6)		3 (9.4)	23 (71.9)	6 (18.8)		30 (93.8)	2 (6.3)	
SDF1-3′A (%)	268 (100)	29 (10.8)	239 (89.2)		7 (2.6)	201 (75.0)	60 (22.4)		143 (53.4; 45.2–61.6)	125 (46.6; 37.9–55.3)	
wt/wt	154 (57.5)	16 (10.4)	138 (89.6)	0.391	6 (3.9)	114 (74.0)	34 (22.1)	0.609	140 (97.9)	14 (11.2)	**< 0.001**
wt/mt	102 (38.1)	13 (12.7)	89 (87.3)		1 (1.0)	77 (75.5)	24 (23.5)		3 (2.1)	99 (79.2)	
mt/mt	12 (4.5)	0 (0.0)	12 (100)		0 (0.0)	10 (83.3)	2 (16.7)		0 (0.0)	12 (9.6)	

Note: wt, wild-type allele; mt, mutant allele. Bold numbers indicate statistical significance for the Chi-square (*p* < 0.05)

### Determinants related to CCR2-64I and SDF1-3’A in HIV-infected individuals

The determinants related to the distribution of polymorphisms in HIV-positive individuals are presented in Table 2. The frequency of CCR2-64I polymorphism was 78% (96/123) wt/wt, 20.3% (25/123) wt/mt, and 1.6% (2/123) mt/mt. A statistically significant relationship was observed between ABO blood groups with the distribution of CCR2-64I polymorphism, with 50% of the polymorphism in the homozygous mutant form (mt/mt) observed in AB and O blood groups simultaneously (*p* = 0.006). Furthermore, the distribution of HIV-1 subtypes into HIV-1 C and non-C was statistically related to the presence of the CCR2-64I polymorphism, with the HIV-1 C subtype being prevalent in heterozygous patients (wt/mt). In contrast, HIV-1 non-C subtypes were more frequent in homozygous patients with the presence of the allele (mt/mt) (*p* = 0.015). On the other hand, the frequency of SDF1-3 A polymorphism was 11.2% (14/125) wt/wt, 79.2% (99/125) wt/mt, and 9.6% (12/125) mt/mt. A statistically significant relationship was observed between age groups with the distribution of SDF1-3 A polymorphism, with 83.3% of the polymorphism in the homozygous mutant form (mt/mt) observed in the age group of 20 to 40 years (*p* = 0.024). Furthermore, the distribution of the RH blood group was statistically related to the presence of the SDF1-3 A polymorphism, with RH + being prevalent whether in heterozygotes (wt/mt) or homozygotes with the presence of the allele (mt/mt) (*p* = 0.018).

**Table 2:** Demographic, biological, and virologic determinants related to the distribution of host molecular factors CCR2-64I and SDF1-3’A in HIV-infected individuals in Luanda, Angola

Independent variables	**Overall (%)**	CCR2-64I				SDF1-3′A				
		wt/wt (%)	wt/mt (%)	mt/mt (%)	p-value	Overall (%)	wt/wt (%)	wt/mt (%)	mt/mt (%)	*p*-value
Overall	123 (100)	96 (78.0)	25 (20.3)	2 (1.6)		125 (100)	14 (11.2)	99 (79.2)	12 (9.6)	
Age group (years)										
<20yrs	3 (2.4)	1 (1.0)	2 (8.0)	0 (0.0)	0.27	3 (2.4)	2 (14.3)	1 (1.0)	0 (0.0)	**0.024**
20-40yrs	93 (75.6)	72 (75.0)	19 (76.0)	2 (100)		95 (76.0)	11 (78.6)	74 (74.7)	10 (83.3)	
>40yrs	27 (22.0)	23 (24.0)	4 (16.0)	0 (0.0)		27 (21.6)	1 (7.1)	24 (24.2)	2 (16.7)	
Gender										
Female	14 (11.4)	12 (12.5)	2 (8.0)	0 (0.0)	0.719	14 (11.2)	1 (7.1)	13 (13.1)	0 (0.0)	0.347
Male	109 (88.6)	84 (87.5)	23 (92.0)	2 (100)		111 (88.8)	13 (92.9)	86 (86.9)	12 (100)	
ABO blood group										
A	31 (25.2)	29 (30.2)	2 (8.0)	0 (0.0)	**0.006**	31 (24.8)	1 (7.1)	28 (28.3)	2 (16.7)	0.337
B	27 (22.0)	20 (20.8)	7 (28.0)	0 (0.0)		28 (22.4)	6 (42.9)	19 (19.2)	3 (25.0)	
AB	5 (4.1)	2 (2.1)	2 (8.0)	1 (50.0)		5 (4.0)	1 (7.1)	4 (4.0)	0 (0.0)	
O	60 (48.8)	45 (46.9)	14 (56.0)	1 (50.0)		61 (48.8)	6 (42.9)	48 (48.5)	7 (58.3)	
RH group										
RH-	1 (0.8)	0 (0.0)	1 (4.0)	0 (0.0)	0.139	1 (0.8)	1 (7.1)	0 (0.0)	0 (0.0)	**0.018**
RH+	122 (99.2)	96 (100)	24 (96.0)	2 (100)		124 (99.2)	13 (92.9)	99 (100)	12 (12)	
HIV-1 subtype										
HIV-1C	7 (22.6)	3 (12.5)	4 (66.7)	0 (0.0)	**0.015**	7 (22.6)	3 (60.0)	4 (17.4)	0 (0.0)	0.073
HIV-1 non-C	24 (77.4)	21 (87.5)	2 (33.3)	1 (100)		24 (77.4)	2 (40.0)	19 (82.6)	3 (100)	
Transmitted drug resistance										
No	22 (78.6)	17 (77.3)	4 (80.0)	1 (100)	0.86	22 (78.6)	4 (80.0)	15 (75.0)	3 (100)	0.614
Yes	6 (21.4)	5 (22.7)	1 (20.0)	0 (0.0)		6 (21.4)	1 (20.0)	5 (25.0)	0 (0.0)	
Viral load (Log_10_)										
<4	5 (21.7)	4 (23.5)	1 (20.0)	0 (0.0)	0.352	5 (21.7)	0 (0.0)	5 (31.3)	0 (0.0)	0.156
4–5	10 (43.5)	9 (52.9)	1 (20.0)	0 (0.0)		10 (43.5)	1 (25.0)	8 (50.0)	1 (33.3)	
>5	8 (34.8)	4 (23.5)	3 (60.0)	1 (100)		8 (34.8)	3 (75.0)	3 (18.8)	2 (66.7)	

Note: wt, wild-type allele; mt, mutant allele. Bold numbers indicate statistical significance for the Chi-square (*p* < 0.05)

## Discussion

To the best of our knowledge, this was the first study to describe the distribution of CCR5-Delta32, CCR2-64I, and SDF1-3′A in the Angolan population as well as their relationship to HIV infection and progression to AIDS in Angola. Our analysis revealed the absence of the CCR5-Delta32 mutation in both HIV-negative and HIV-positive individuals in Luanda, the capital city of Angola, which might be a reflection of this allele frequency in the general population. Indeed, this mutation has been reported to exist principally at appreciable frequencies in Europe and western Asia but is low or even absent in Asians and/or Africans, which is consistent with our findings [23–25].

The CCR2-64I is common in most ethnic groups, including the African population [26]. A frequency of 60.2% of the CCR2-64I mutation was found in the present study, which was high compared to that reported in African regions, around 31% [27–29]. Our results showed that the CCR2-64I mutation has a possible protective effect on HIV transmission in the Angolan population (*p* < 0.001), with the heterozygous form (wt/mt) present in 80% of uninfected individuals compared to around 20% for infected individuals, while in the homozygous form (mt/mt), the mutation was present in 94% of uninfected individuals compared to 6% for infected individuals (Table 1), showing that the presence of the mutation may be playing an important role in the susceptibility to HIV infection in this population. Indeed, it was observed that 94% of wild-type individuals (wt/wt) had HIV infection compared to only 6% of wild-type individuals who did not have the infection. Furthermore, the CCR2-64I mutation was related to the ABO blood group, with individuals in groups AB and O being the most susceptible to presenting the mutation (*p* = 0.006). Regarding HIV-1 subtypes, our findings indicate a statistically significant relationship (*p* = 0.015) between the CCR2-64I mutation in the heterozygous form (wt/mt) with HIV-1-C (67%), while the homozygous form (mt/mt) with non-C subtypes (100%). It is also worth mentioning that, although without statistical significance (*p* > 0.05), 20% of the individuals who presented some transmitted resistance mutation, whether conferring resistance against PR, RT, or IN, presented the CCR2-64I mutation in the heterozygous form (wt/mt) (Table 2). Also, high viral load values (> 5Log_10_) were observed among HIV patients with heterozygous (60%) or homozygous (100%). Therefore, our results are consistent with the hypothesis that CCR2-64I alleles can delay HIV disease progression [27] without affecting susceptibility to infection, which agrees with what was reported by Ma et al., 2005 [29]. Contrary to what was observed in our study, a meta-analysis based on published studies on CCR2-64I regarding susceptibility to HIV infection concluded that this allele does not affect the risk of HIV-1 infection [30]. This discrepancy in the scientific literature shows that further studies should be conducted to verify the role of the CCR2-64I mutation in susceptibility to HIV infection, AIDS progression, and/or mortality in people living with HIV in Africa.

We observed a frequency of 43% of the SDF1-3 A mutation, below that observed worldwide, around 71%, but it is higher than that reported in other African countries, around 9% [31]. Also, our result is below that observed in the West Region of Cameroon (100%) [22]. Our results showed that the SDF1-3’A mutation has an effect on HIV transmission in the Angolan population (*p* < 0.001), with the heterozygous form (wt/mt) increasing the frequency from 2 to 79% in uninfected individuals to infected individuals, respectively, while in the homozygous form (mt/mt) it increases from 0 to 10% in uninfected individuals to infected individuals, respectively (Table 1). Furthermore, although not statistically significant, our findings seem to indicate a relationship between the SDF1-3 A mutation and non-C HIV-1 subtypes, since they were present in 83% of the heterozygous form (wt/mt) and 100% in the homozygous form (mt/mt). It is also worth mentioning that 25% of the individuals who presented some transmitted resistance mutation, whether conferring resistance against PR, RT, or IN, presented the SDF1-3 A mutation in the heterozygous form (wt/mt).

Also, high viral load values (> 5Log_10_) were observed among HIV patients with wild-type (75%) compared to heterozygous (19%) or homozygous (67%) patients (Table 2). Our results differ from those observed by Ma et al., 2005, who reported that SDF1-3’A did not differ between HIV-seropositive and HIV-seronegative individuals from Cameroon [29].

Our study highlights the possible genetic factors present in the population that have played a crucial role in the susceptibility and progression of AIDS. However, the study does not present data on the relationship of these CCR2-64I and SDF1-3 A mutations with mortality, clinical profile or distribution of defence cells, or other important biological functions that define AIDS. The three genes (CCR5-Delta32, CCR2-64I, and SDF1-3′A) were not typed in all 284 participants due to technical challenges encountered during the assays, such as poor DNA quality or insufficient DNA quantity in some samples, which limited the successful amplification and analysis of certain genetic markers, despite repeated attempts. It is worth mentioning that several factors, such as host susceptibility, genetics and immune function, access to health care, coinfections, nutritional status, and viral genetic variability, can affect the susceptibility to HIV infection as well as the rate of progression and death from AIDS [32]. Therefore, future studies to analyse these distinct biological and non-biological factors that may explain the susceptibility to HIV infection and AIDS progression should be conducted with a large sample size in the Angolan population.

## Conclusion

We investigated CCR5-Delta32, CCR2-64I, and SDF1-3’A genetic polymorphisms in HIV-infected and uninfected individuals from Angola, a sub-Saharan African country. As expected, we found no CCR5-Delta32 mutation. On the other hand, CCR2-64I and SDF1-3’A were found and presented a relationship with HIV infection, age, ABO/Rh blood group, and HIV-1 subtypes. Further studies investigating biological and non-biological factors related to susceptibility to HIV infection and AIDS progression or death should be conducted in Angola.

## Data Availability

The datasets used and/or analysed during the current study are available from the corresponding author on reasonable request.
